# Superior Transverse Atraumatic Reconstruction (STAR) approach provides a better-compared outcome to standard Direct Superior Approach (DSA): a matched, prospective comparative single-surgeon study

**DOI:** 10.1051/sicotj/2023008

**Published:** 2023-04-21

**Authors:** Eustathios Kenanidis, Nikolaos Milonakis, Foukarakis Georgios, Michael Potoupnis, Eleftherios Tsiridis

**Affiliations:** 1 Academic Orthopaedic Department, Aristotle University Medical School, General Hospital Papageorgiou Ring Road Efkarpia Thessaloniki 56403 Greece; 2 Centre of Orthopaedic and Regenerative Medicine (CORE), Center for Interdisciplinary Research and Innovation (CIRI)-Aristotle University of Thessaloniki (AUTH), Balkan Center, Buildings A & B, Thessaloniki 10th km Thessaloniki-Thermi Rd P.O. Box 8318 GR 57001 Greece; 3 Tsiridis Orthopaedic Institute – ICAROS Clinic Thessaloniki Greece

**Keywords:** STAR approach, DSA approach, THA, Total hip arthroplasty, Minimal invasive surgery, STAR

## Abstract

*Introduction*: The Direct Superior Approach (DSA) is a muscle-sparing hip approach that does not protect the piriformis and the other short external rotators. We present a DSA modification we named STAR (Superior Transverse Atraumatic Reconstruction), which has DSA advantages but always preserves piriformis. Our study compared the early postoperative, radiological, and functional results of patients undergoing primary total hip arthroplasty (THA) through the STAR approach with a matched DSA group performed by a senior surgeon. *Methods*: Each group, DSA, and STAR included 200 elective primary unilateral THAs performed by the surgeon between 2016–2017 and 2020–2021, respectively. Patients were included in both groups using the same inclusion criteria. Both groups were matched for age and sex. The same postoperative pain management, chemoprophylaxis, and physiotherapy protocols were followed in both groups. Two independent orthopaedic surgeons performed the clinical and radiological follow-up. *Results*: The STAR group had significantly lower mean incision length (*p* = 0.042) and hospital stay (*p* = 0.002) than the DSA group. The mean intraoperative blood loss (*p* = 0.085) and the need for blood transfusion (*p* = 0.228) were less for the STAR than the DSA group. The mean postoperative functional scores improvement was significantly higher for the STAR than the DSA group at the end of the first and third postoperative months. *Conclusions*: The STAR approach offers earlier functional improvement, shorter hospital stay and less transfusion need than DSA for patients undergoing primary THA. Both approaches showed a limited complication risk and an outstanding acetabular and femoral access enabling the procedure.

## Introduction

The ideal hip approach for total hip arthroplasty (THA) should be simple, muscle-sparing, providing unimpeded exposure and rapid, painless recovery with a minimal hospital stay [1–3]. The Direct Superior approach (DSA) is a minimally invasive (MIS) hip approach, protecting the iliotibial band and quadratus femoris but not the piriformis (PF) and the other short external rotators (SERs) [2, 4]. DSA provides exceptional acetabular and femoral access for standard and complex primary hips using standard instrumentation [2, 3]. It is a quickly learned, uncomplicated approach to delivering early postoperative rehabilitation and excellent outcomes [5–9].

We present a DSA modification, which we name STAR standing for Superior Transverse Atraumatic Reconstruction approach, which in addition to the other DSA advantages, always preserves PF. We hypothesize that PF preservation is essential to enhance hip stability, early and safe rehabilitation and functional outcomes. Our study aimed to compare the early postoperative, radiological and functional results and PROMs between patients undergoing primary THA for hip osteoarthritis through the STAR approach with a matched DSA control group, using the same protocol and implants and performed by the same senior surgeon.

## Methods

A senior surgeon performed 200 elective primary unilateral THAs through the STAR approach (STAR group) between May 2020 and September 2021. Adult patients with primary or complex hip osteoarthritis and the American Society of Anaesthesiologists (ASA) score ≤ 3 were included in the study. All patients were preoperatively informed about the hip approach type that would be used. Patients with a malignant tumour, posttraumatic or severely dysplastic hips, and revision THAs were excluded from the study.

The DSA group was a 200 THAs historical cohort performed by the senior surgeon between 2016 and 2017 [3]. Patients were included in the DSA group using the same criteria as the STAR group. Frequency matching was used to match both groups for age and sex. The same standard intraoperative surgical instrumentation, pain management, chemoprophylaxis and physiotherapy protocols were used for both groups’ patients. Two independent fellowship-trained consultant orthopaedic surgeons, not involved in the surgical procedures, performed the clinical and radiological follow-up (preoperatively, one, three and twelve months postoperatively).

### Operative techniques

**Figure 1:**
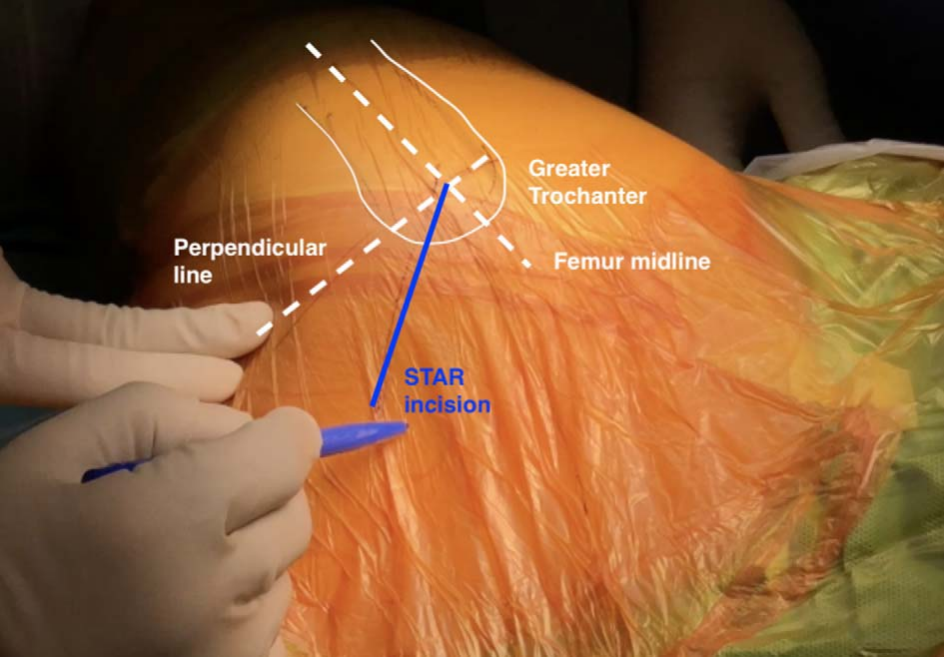
The STAR incision starts at the crossing of the femur’s midline with a perpendicular line two centimetres below the tip of the greater trochanter.

All patients were placed in the lateral decubitus position. The DSA approach has been previously described [3]. The STAR is a DSA approach modification. We here describe in detail the different steps of the STAR approach. Concerning the skin incision, the surgeon should palpate and draw the greater trochanter tip and proximal femur, indicating the anterior and posterior femoral cortexes (Figure 1). Aiming to recognize the piriformis fossa level, we divide the greater trochanter and proximal femoral area into anterior and posterior halves. A point 2 cm below the greater trochanter tip on the femur’s midline is identified, and at this level, a perpendicular line to the femoral midline is drawn. The crossing of the two lines indicates the approach starting point. The 8–10 cm skin incision is drawn 45° posteriorly and upwards from the perpendicular line (Figure 1). The gluteus maximus muscle fibres are then easily split apart. Following the SERs and sciatic nerve exposure, hemostasis is meticulously performed at the SERs’ femoral insertion by easily coagulating the apparent medial circumflex femoral artery (MCFA) branches.

**Figure 2:**
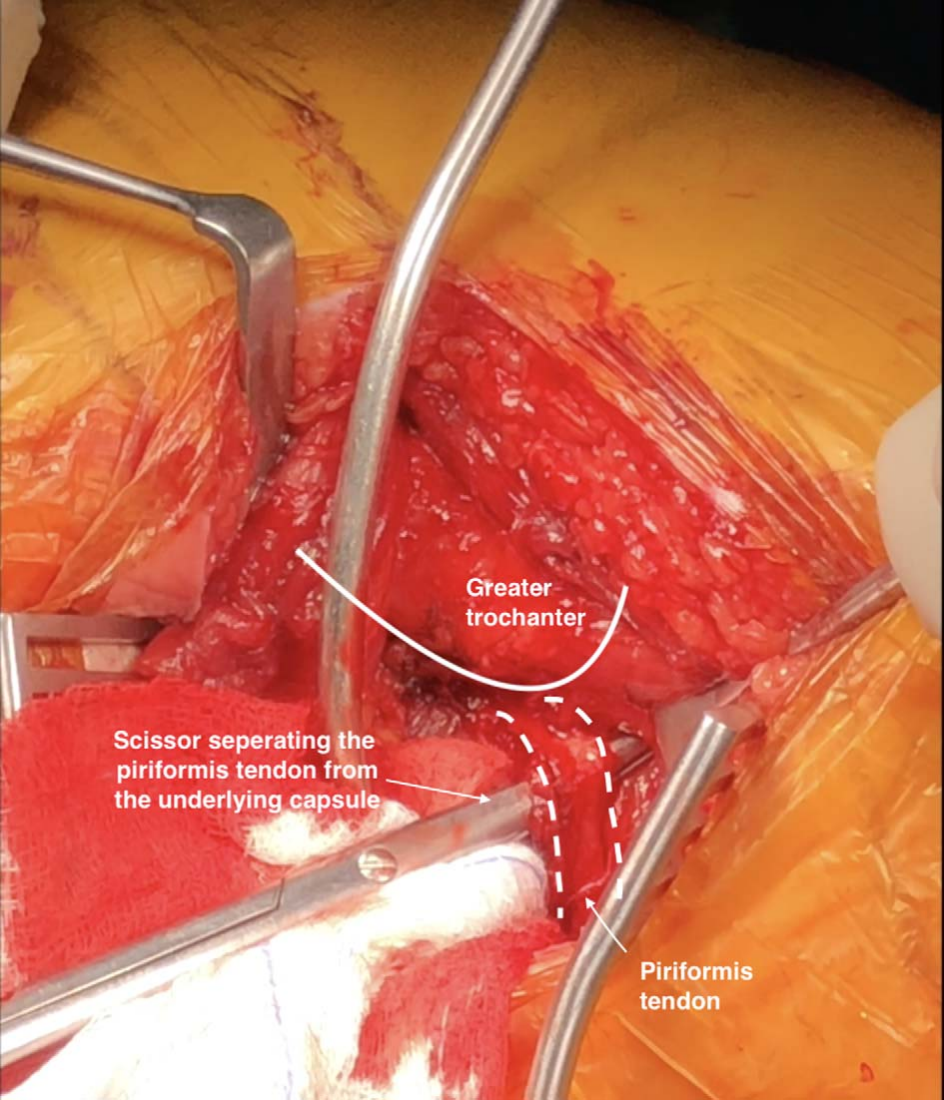
The figure depicts the separation of the piriformis tendon from the underlying capsule using a scissor.

The surgeon then recognizes and places a Langenbeck retractor under the gluteus medius (GMed) to reveal the PF and gluteus minimus (GMin) muscles effectively. The interval between PF and the other SERs is then recognized, and the PF is bluntly separated from the underlying capsule using a scissor with the hip in slight abduction, and it is retracted cranially and upwards with a small, curved retractor along with GMin (Figure 2). Following hip internal rotation and slight extension so as not to stretch the PF, the obturator internus and gemelli tendons are tenotomised close to their femoral insertion, stripped off the posterior capsule, tagged with Ethibond suture 5/0, and retracted posteriorly to protect the sciatic nerve. The capsule is then incised in an “inverted J” from the quadratus femoris border to the PF southern border down to the acetabulum, tagged with Ethibond 5/0 and extroverted over the SERs flap to protect the sciatic nerve. During acetabular preparation, the PF has positioned away from the plane as the proximal femur is retracted anteriorly with the retractor placed over the anterior acetabular rim. During femoral preparation, the femur is easily exposed by lifting it from behind its posterior aspect, and as the leg is turned in adduction, flexion and internal rotation, the whole proximal femoral view becomes available. The assistant surgeon should always exert longitudinal force on the leg towards the surgeon to adequately expose the femur and minimize the PF stretching. The PF is always palpable and protected during broaching and stem insertion. The capsule and the SERs are repaired back to their anatomical position through drilled bone holes.

### Perioperative management

All patients received general anaesthesia. Chemoprophylaxis began with one dose preoperatively and continued for 24 h postoperatively. The STAR group received intravenous teicoplanin 400 mg twice a day and cefuroxime 750 mg three times a day, and the DSA group received vancomycin 500 mg twice a day and the same cefuroxime dose. One tranexamic acid gram was given intravenously preoperatively for all patients. Low-molecular-weight heparin or rivaroxaban postoperatively continued daily for a month. Intravenous 1 g paracetamol three times, lornoxicam 8 mg twice a day and tramadol 100 mg as required for 24–36 h were used to control postoperative pain for both groups. Patients were discharged home on paracetamol and lornoxicam for 1–2 weeks.

### Preoperative and postoperative assessment

A comprehensive medical history preoperatively and other intra- and postoperative parameters, including the incision length, operative time, implant type, anaesthetic technique, blood loss, transfusion rate, complications, hospital stay length (LOS), re-admissions and revision rates, were documented. LOS also included the admission day. All patients were followed up clinically (Harris Hip Score/HHS and Hip Disability and Osteoarthritis Outcome Score/HOOS) and radiologically (cup inclination and stem coronal alignment) preoperatively and during the first postoperative year (1, 3, and 12 months).

### Statistical analysis

Statistical tests were two-tailed; *p*-values < 0.05 were counted as statistically significant. Standard statistical methods were used for descriptive statistics. Kolmogorov-Smirnov and Shapiro-Wilk tests evaluated data distribution normality. Normally and not normally distributed continuous variables were compared using a two-sided independent sample t-test and the Mann-Whitney U-test, respectively. Categorical variables were evaluated using the Chi-square test. Cohen kappa coefficient (*κ*) was used to assess interrater agreement between raters. Statistical analyses were accomplished using SPSS software (IBM, version 25.0).

## Results

Two hundred patients were enrolled, and no patient was lost to follow-up from the STAR group. The DSA group comprised 204 patients previously performed, and four patients were lost to follow-up from this historical group [3].

Demographics and baseline patient characteristics are depicted in Table 1. Both groups were matched for age and sex. Besides, the ASA score, preoperative diagnosis and the mean BMI were comparable between groups. Perioperative patients’ data and implant characteristics are depicted in Table 2. Hybrid THA (Trident cup/Exeter stem, Stryker, Mahwah, USA) and uncemented THAs (Pinnacle cup/Corail stem, Depuy Synthes and RM Monoblock cup/twinSys stem, Matthys European Orthopaedics) were used in both groups. The cementless and hybrid THAs percentage was similar between groups (*p* = 0.92) (Table 2).

**Table 1:** The demographics and preoperative baseline characteristics of both groups’ patients.

Parameters		STAR group	DSA group	*p*
Number**		200	200	–
Age (years)*		67.66 ± 7.76 (50–88)	66.53 ± 8.87 (49–87)	0.207@
Sex***	Male	84 (42)	71 (35.5)	0.182#
	Female	116 (58)	129 (64.5)	
BMI (kg/m^2^)*		27.36 ± 2.7	27.59 ± 2.98	0.523@
		(22–38.8)	(22–39.7)	
BMI less than 30 kgr/m^2^ ***		165 (82.5)	154 (77)	0.171#
BMI more than 30 kgr/m^2^		35 (17.5)	46 (23)	
ASA grade***	I	79 (39.5)	62 (31)	0.134#
	II	105 (52.5)	114 (57)	
	III	16 (8)	24 (12)	
Operated side***	Right	134 (67)	119 (59.5)	0.120#
	Left	66 (33)	81 (40.5)	
Preoperative diagnosis***				
Primary osteoarthritis		142 (71)	135 (67.5)	0.133#
Hip Dysplasia Hartofylakidis type I		20 (10)	29 (14.5)	
Hip Dysplasia Hartofylakidis type II		20 (10)	19 (9.5)	
Avascular necrosis		14 (7)	6 (3)	
Rheumatoid arthritis		3 (1.5)	7 (3.5)	
Psoriatic arthritis		1 (0.5)	4 (2)	

* The values are given as the mean with the standard deviation (±) and the range in parentheses.

** The values are given as raw numbers.

*** The values are given as raw numbers with the percentages in parentheses.

@ Tests were performed using the Mann–Whitney test.

# Tests were performed using χ^2^ test.

BMI = body mass index, ASA = American Society of Anesthesiologists score.

**Table 2:** Intraoperative and postoperative clinical and radiological data of both groups.

Operative and radiological data		STAR group	DSA group	*p*
Incision length (cm)*		9.02 ± 1.47 (7–14)	9.15 ± 1.32 (8–14)	0.042@
Operation time (min)*		60.4 ± 12.54 (40–95)	59.35 ± 13.37 (45–95)	0.113@
Estimated intraoperative blood loss (mL)*		177.05 ± 77.75 (40–360)	191.2 ± 80.86 (50–450)	0.085@
Blood transfusion**	Yes	29 (14.5)	38 (19)	0.228#
	No	171 (85.5)	162 (81)	
Hospital stay (days)*		2.35 ± 0.56 (2–4)	2.53 ± 0.64 (2–4)	0.002@
Discharge**	Home	181 (90.5)	184 (92)	0.596#
	Rehabilitation	19 (9.5)	16 (8)	
Cup type***	Trident	91	92	0.077#
	Pinnacle	72	55	
	RM	37	53	
Acetabular cup diameter*		51.46 ± 1.87	50.53 ± 3.14 (46–58)	<0.001@
Screws for cup fixation*		1.6 ± 0.49 (1–2)	1.54 ± 0.81 (0–3)	0.141@
Bearing type**	MoP	26 (13)	36 (18)	0.167#
	CoP	174 (87)	164 (82)	
Head diameter (mm)**	32	19 (9.5)	85 (42.5)	<0.001@
	36	181 (90.5)	115 (57.5)	
Cup orientation*	Inclination	44.35 ± 3.15 (34–50)	44.15 ± 3.35 (31–49)	0.743@
Stem coronal alignment**	Neutral	196 (98)	194 (97)	0.522#
	Varus	4 (2)	6 (3)	
	Valgus	0 (0)	0 (0)	

* The values are given as the mean with the standard deviation (±) and range in parentheses.

** The values are given as raw numbers with the percentages in parentheses.

*** The values are given as raw numbers.

@ Tests were performed using the Mann–Whitney test.

# Tests were performed using χ^2^ test.

MoP (Metal on Polyethylene), CoP (Ceramic on Polyethylene).

The mean incision length (*p* = 0.042) and the mean LOS (*p* = 0.002) were significantly lower for the STAR than the DSA group. The mean estimated intraoperative blood loss (*p* = 0.085) and the percentage of patients needing blood transfusion (*p* = 0.228) were non-significantly fewer for the STAR than the DSA group. The mean operation time (*p* = 0.113), the cup screw number (*p* = 0.141), the bearing type (*p* = 0.167), and the mean cup inclination and stem coronal alignment did not differ between groups. Significantly more 36 mm heads were used in the STAR than in the DSA group (*p* = 0.001) (Table 2).

No serious adverse events, such as perioperative fractures, sciatic nerve lesions, thromboembolic events, hip dislocation and acute deep infection cases, were recorded in either of the two groups. Three superficial wound infections were managed with oral antibiotics in overweight patients, two in STAR and one in the DSA group. A mild wound bruising or limited hematoma was noted in eight DSA and seven STAR group patients (*p* = 0.792) and healed spontaneously during the first postoperative days.

Preoperatively, the mean HHS and HOOS symptoms and pain subscores were significantly better for the DSA group. All the other HOOS subscores were preoperatively comparable between the groups (Table 3). Postoperatively, both groups demonstrated a significant improvement in the mean HHS and HOOS scores at all follow-up times than the preoperative mean scores. However, the mean HHS and HOOS symptoms, pain and ADL subscores on the first postoperative month and the mean HOOS QQL score on the third postoperative month were significantly better for the STAR than the DSA group. No other functional scores differences among groups were recorded till the first postoperative year (Table 3). A strong agreement between raters, *κ* > 0.8, *p* < 0.001, was observed for all parameters screened.

**Table 3:** Preoperative and postoperative outcomes data given as mean ± standard deviation.

	DSA group	STAR group	*p*
HHS			
Preoperative	44.79 ± 5.0	42.34 ± 4.58	<0.001
1 month	79.99 ± 4.64	80.22 ± 6.31	0.048
3 months	87.94 ± 5.0	88.15 ± 5.18	0.258
12 months	91.45 ± 5.38	92.34 ± 3.65	0.122
HOOS			
Symptoms			
Preoperative	44.89 ± 5.72	43.4 ± 5.59	0.025
1 month	74.93 ± 5.57	75.97 ± 6.58	0.041
3 months	87.95 ± 5.44	88.37 ± 5.03	0.49
12 months	91.89 ± 5.63	92.57 ± 4.48	0.117
Pain			
Preoperative	41.38 ± 5.02	38.5 ± 6.56	0.001
1 month	78.85 ± 5.48	79.41 ± 6.02	0.030
3 months	88.23 ± 5.63	88.72 ± 5.63	0.146
12 months	92.01 ± 5.79	92.53 ± 3.53	0.987
ADL			
Preoperative	37.90 ± 5.15	37.61 ± 5.36	0.744
1 month	79.29 ± 6.02	79.94 ± 7.49	0.022
3 months	87.47 ± 5.86	87.69 ± 6.10	0.175
12 months	92.16 ± 6.34	92.58 ± 5.06	0.551
S&R			
Preoperative	37.26 ± 11.42	35.33 ± 11.22	0.787
1 month	43.96 ± 14.38	41.11 ± 11.65	0.193
3 months	55.50 ± 17.31	54.33 ± 14.12	0.651
12 months	72.35 ± 21.29	71.96 ± 17.86	0.724
QQL			
Preoperative	39.69 ± 11.79	39.46 ± 8.05	0.130
1 month	53.06 ± 13.33	53.96 ± 9.04	0.139
3 months	66.87 ± 12.76	68.52 ± 8.37	0.042
12 months	82.99 ± 12.31	83.77 ± 8.09	0.177

HHS: Harris Hip Score, HOOS: Hip Disability and Arthritis Outcomes Score, ADL: Activities of Daily Living, S&R: Sport & Recreation, QOL: Quality of Life.

## Discussion

This study evaluated the perioperative, early postoperative functional and radiological results of two matched groups of patients undertaking primary THA through the STAR or DSA approaches. The same high-volume surgeon performed all procedures using similar perioperative and postoperative patient management and implants. PF tendon preservation was the principal difference between the groups. We concluded that the STAR approach is related to significantly better patients’ functional scores, less pain during the first three postoperative months, and shorter LOS than DSA. Complications rate, surgical time and radiological data were similar among groups.

### Functional results

Our chief finding was the patients’ earlier functional improvement undergoing primary THA through the STAR than the DSA approach that was primarily attributed to preserving PF muscle. Both approaches are tissue-preserving, facilitating early functional patient improvement. The iliotibial band and quadratus femoris preservation, the smaller incision, the SERs and capsule repair [2, 8, 9], the surgeon’s seniority, physiotherapy and postoperative pain management protocols may have contributed to the early functional recovery of both groups; however, these parameters were similar between groups and cannot be considered responsible for their different outcomes. A recent posterior approaches’ comparative study found that the PF muscle demonstrated significantly less contiguity, atrophy and better function in patients where the PF was preserved than in those where PF was reattached [10]. Another RCT comparing PF preservation or re-attachment after standard posterior approach THA demonstrated significantly less muscle grade and bulk deterioration in the PF-preserving group at three postoperative months but no functional differences at two postoperative years [11]. A radiological study also supported that PF preservation resulted in no significant muscle fatty infiltration in the third postoperative month [12]. Our outcomes also support that PF preservation was the main reason for the early functional differences between groups.

### Early discharge

Our study demonstrated that the STAR mean LOS was significantly shorter than the DSA group. Our registry data record LOS from admission to discharge. Our practice admits patients the day before for preop assessment and anaesthetic review. The surgery day is the following morning; therefore, at a minimum, the actual LOS is 20 h less than the recorded timescale. Both approaches facilitated immediate patient mobilization allowing early discharge from the hospital. In our study, the significant LOS difference between groups can be attributed to the earlier functional improvement, the fewer blood loss and transfusion rates and the significantly smaller wound incision length of the STAR than the DSA group.

### Accessibility, efficacy and safety

MIS approaches have been associated with a higher complication risk due to obstructed access and the prolonged learning curve [13–15]. STAR and DSA approaches have an outstanding acetabular and femoral view, needing standard instruments when using any implant [2, 9]. Siddappa and Meftah supported that highly reproducible results can be achieved with PF preservation; however, the superior acetabular part visualization may be hampered by needing capsular releases and femoral placement adjustments to achieve proper acetabular access [16]. In our experience, the PF tendon did not impede the acetabular or femoral visualization in any THA. A key point is the initial tendinous and distal muscular PF part release from the posterior capsule that sets the muscle free and allows better mobilization. During acetabular reaming, the retractor over the anterior acetabular wall moves the femur and PF muscle anteriorly and superiorly from the surgical field. Concerning the femoral side, the PF tendon usually attaches to the greater trochanter medial aspect and the joint capsule superior to the trochanteric fossa, away from the intramedullary broaching entry point during THR [17]. The longitudinal force that the assistant surgeon exerts on the leg towards the surgeon further relaxes and removes the PF muscle away from the surgical field. The tendon mobilization is only tricky for dysplastic hips with short offset and long-lasting arthritis, probably due to the muscle elasticity loss. This elasticity loss may impede the SERs’ detachment or overstretch the muscles and destroy them intraoperatively [18].

### Complications

The complication rate was minimal and comparable between the groups. Sciatic nerve lesions were not documented, and wound complications incidence was very low, which can be attributed to the surgeon’s seniority, the limited operative time and the unobstructed view-making efficiently performed procedures. No dislocations were also documented for both group. During 90° hip flexion, the PF lies posteriorly to the hip and is considered a critical hip joint stabilizer [17]. In our study, the STAR group had a significantly higher number of 36 mm heads involved than the DSA group, but this did not make any difference. The surgeon’s seniority, the unimpeded access, the implantation accuracy, the PF preservation and the large femoral heads (32/36 mm) explain the dislocations’ absence [19].

### Blood loss

Blood transfusions were less needed for the STAR than for the DSA group. DSA and STAR are soft-tissue-friendly approaches with limited blood loss as they are advantageously away from critical vessels [2]. The chief blood supply that crosses the approach field comes from the MFCA branches that can be effortlessly found and cauterized [20]. The lower mean blood loss for the STAR group can be partly explained by the PF preservation and the shorter incision compared to the DSA approach.

### Study’s limitations

Our study’s first limitation is that we used a historical control group to compare with the study group; no randomization was performed, which may generate some selection bias. Secondly, we assessed the short-term patients’ results; however, one-year postoperative time is adequate to evaluate the approach performance. Our study’s advantages include the senior surgeon’s involvement in performing all procedures using the same implants, the groups matching for demographics and comparability for numerous intraoperative and postoperative parameters and that the outcomes were recorded by physicians not involved in any case.

## Conclusions

Our study demonstrated that the STAR approach facilitated earlier functional improvement, shorter hospital stays, better cosmetic outcomes, less transfusion need, a similar limited complication risk profile, and outstanding acetabular and femoral access for patients undergoing primary THA compared to the DSA approach.
