# Correction Notice to: Superior Transverse Atraumatic Reconstruction (STAR) approach provides a better-compared outcome to standard Direct Superior Approach (DSA): a matched, prospective comparative single-surgeon study

**DOI:** 10.1051/sicotj/2023021

**Published:** 2023-07-21

**Authors:** Eustathios Kenanidis, Nikolaos Milonakis, Georgios Foukarakis, Michael Potoupnis, Eleftherios Tsiridis

**Affiliations:** 1 Academic Orthopaedic Department, Aristotle University Medical School, General Hospital Papageorgiou Ring Road Efkarpia Thessaloniki 56403 Greece; 2 Centre of Orthopaedic and Regenerative Medicine (CORE), Center for Interdisciplinary Research and Innovation (CIRI)-Aristotle University of Thessaloniki (AUTH), Balkan Center Buildings A & B, 10th km Thessaloniki-Thermi Rd P.O. Box 8318 Thessaloniki GR 57001 Greece; 3 Tsiridis Orthopaedic Institute – ICAROS Clinic Thessaloniki Greece

The article was published April 21, 2023 with an error in the name of one of the co-author.

The full name of this co-author is: Georgios Foukarakis.

